# Temperature-Induced Seasonal Dynamics of Brain Gangliosides in Rainbow Trout (*Oncorhynchus mykiss* Walbaum) and Common Carp (*Cyprinus carpio* L.)

**DOI:** 10.3390/life14101273

**Published:** 2024-10-07

**Authors:** Valentina Pavić, Barbara Viljetić, Senka Blažetić, Irena Labak, Elizabeta Has-Schön, Marija Heffer

**Affiliations:** 1 Department of Biology, Josip Juraj Strossmayer University of Osijek, Ulica cara Hadrijana 8A, 31000 Osijek, Croatia; vpavic@biologija.unios.hr (V.P.); senka@biologija.unios.hr (S.B.); hasschon.elizabeta@gmail.com (E.H.-S.); 2 Department of Chemistry, Biochemistry and Clinical Chemistry, School of Medicine, Josip Juraj Strossmayer University of Osijek, 31000 Osijek, Croatia; bviljetic@mefos.hr; 3 Department of Medical Biology, School of Medicine, Josip Juraj Strossmayer University of Osijek, 31000 Osijek, Croatia; mheffer@mefos.hr

**Keywords:** poikilothermic species, environmental stimuli, sialic acid, immunohistochemistry, thin-layer chromatography

## Abstract

This study aimed to determine the expression and distribution of gangliosides in specific regions of the brains of rainbow trout (*Oncorhynchus mykiss* Walbaum) and common carp (*Cyprinus carpio* L.) with regard to seasonal temperature changes. Seasonal changes in ganglioside expression and distribution within the species were expected. The natural ecosystems of these fishes differ significantly due to their distinct habitat preferences, geographic distributions, and environmental requirements. Based on the fact that the common carp is eurythermic and adapts to a wide range of temperatures, while the rainbow trout is stenothermic and thrives in a narrower temperature range, it was expected that these species would exhibit distinct patterns of ganglioside modification as part of their adaptive response to temperature fluctuations. Immunohistochemistry using specific antibodies for the major brain gangliosides (GM1, GD1a, GD1b, GT1b), along with the Svennerholm method for quantifying sialic acid bound to gangliosides, revealed that cold acclimatization led to an increase in polysialylated gangliosides in the common carp brain and an increase in trisialogangliosides in the rainbow trout brain. Immunohistochemical analysis also identified region-specific changes in ganglioside expression, suggesting specific functional roles in neuronal adaptation. These results supported the hypothesis that the composition and distribution of brain gangliosides change in response to seasonal thermal shifts as part of the adaptive response. The results underscore the importance of gangliosides in neuronal function and adaptation to environmental stimuli, with implications for understanding fish resilience to temperature changes. This study offers valuable insights into species’ temperature adaptation, with implications for physiological and ecological management and improved aquaculture practices. Future research could expand the species scale, study molecular mechanisms and regulatory pathways in ganglioside metabolism, and examine ganglioside interactions with membrane proteins and lipids for a deeper understanding of thermal adaptation.

## 1. Introduction

Wildlife populations, including aquatic species, are continually exposed to seasonal environmental changes that affect their physiology and behavior [1]. Teleost fish play vital roles in aquatic ecosystems by contributing to nutrient cycling, energy flow, and biogeochemical processes, such as carbon [2,3,4] and nitrogen [5,6] cycling, which influence nutrient availability and ecosystem productivity. As key predators and prey within aquatic food webs, they help regulate the population sizes of other organisms, maintaining ecological balance [7]. Additionally, many teleost species contribute to habitat formation, such as coral reefs and aquatic vegetation beds [8], which provide shelter and breeding grounds for various species [9]. Some teleost fish also aid in seed dispersal for aquatic plants, supporting plant regeneration [10]. Through these diverse ecological roles, teleost fish enhance biodiversity and resilience, serving as crucial indicators of water quality and ecosystem stability [11].

For teleost fish, seasonal temperature transitions play a crucial role in regulating various physiological processes [12,13], particularly due to their ectothermic nature, where their internal temperature is dictated by the surrounding environment [14]. Fish must adapt to these fluctuations to maintain homeostasis, especially in colder months or during periods of rapid temperature shifts, such as the transition from spring to summer [15]. Fish experience both acute and chronic stress due to temperature fluctuations [16]. Sudden increases in temperature, such as during heatwaves, can trigger immediate stress responses, while constant warming imposes long-term thermal (heat) stress [17]. This sustained exposure can adversely affect their well-being, impacting their survival, growth, and reproductive capabilities [18]. If these stressors continue or worsen, they can lead to population declines or even extinctions, with broader ecological impacts that disrupt essential biological processes [19,20].

To counteract these stresses, fish employ a range of adaptive mechanisms, including alterations in neuronal and cellular processes. Fish brain systems make precise physiological and biochemical adjustments to cope with seasonal temperature fluctuations, preserving homeostasis and survival [21]. These modifications involve changes in neurotransmitter levels, neuronal activity, and membrane structure [22,23,24]. Short-term, frequent, or intense stress from temperature changes depletes energy, harms overall health, weakens the immune system, and increases mortality [25]. Although fish seek optimal temperatures, this may be constrained by environmental and species-specific factors [26,27]. Adaptations in the central nervous system, influenced by membrane properties, are crucial [28], with synaptic plasticity and membrane lipid composition adjusting to ensure proper function and survival in varying thermal environments [29,30,31]. Gangliosides, glycosphingolipids abundant in the nervous system, are particularly sensitive to temperature shifts [32] and play a critical role in maintaining membrane fluidity and stability, enabling synaptic plasticity and signal transduction during thermal stress [33,34]. These adaptive changes are essential for maintaining neurological functions and overall fish survival across varying thermal environments [35]. These amphiphilic glycosphingolipids, characterized by varying numbers of negatively charged sialic acids, are highly concentrated in synaptic membranes [36]. Gangliosides are positioned on the outer surface of the ordered lipid signal domains of neuronal membranes, playing a critical role in creating selectivity in membrane transport and contributing to the spatial organization of cells [37,38,39].

Typical features of the teleost brain include a large hindbrain (*rhombencephalon*—rhom), a large unpaired cerebellum (*cerebellum*—cbl), and two prominent visual regions (*tectum opticum*—tect) located dorsally on the tegmentum (*tegmentum*—teg) of the midbrain (*mesencephalon*—mes). The relatively small and extended forebrain (*telencephalon*—tel) is connected to the midbrain via the interbrain (*diencephalon*—dien), and the relatively large olfactory bulbs (*bulbus olfactorius*—bol) are positioned at the rostral end of the *telencephalon* [40]. The rainbow trout brain is dominated by the relatively large optic tectum (*tectum opticum*), which is a functional part of the executive visual system [41]. In contrast, the common carp brain is specialized for chemoreception (taste and smell). The tectum is as large as the vagal lobe of the hindbrain, which is connected to the palatal organ—the functional part of the taste system. In carp, the facial lobe, which processes sensations from chemoreceptor buds located outside the oral cavity (a combined sense of taste and smell, perception in mammals), is also enlarged [41].

Fish in cold habitats exhibit a predominance of high-polarity gangliosides, which enhance synaptic transmission for cold adaptation [42]. This increases nerve excitability at the cost of membrane stability, leading to greater lipid fluidity and a more disordered brain membrane compared to warmer-adapted fish [43].

Glucosylceramide (GlcCer), a precursor in ganglioside biosynthesis, varies across species, with vertebrate glycosphingolipids (Galβ1-4Glc-Cer) differing significantly from those in other organisms. Galactosylceramide (GalCer) dominates in the deuterostome nervous system, while protostomes feature glucosylceramide, reflecting an evolutionary shift linked to increasing nervous system complexity and myelin development [44]. Studies across 78 vertebrate species show ganglioside rises with nervous system complexity, with cold-blooded vertebrates having more complex, highly sialylated gangliosides in their CNSs and warm-blooded species having fewer, less polar gangliosides. These patterns suggest that gangliosides contributed to brain differentiation and functional evolution [45], with variations in their concentration aiding thermal adaptation by maintaining optimal neuronal transmission, as seen in higher sialylated fractions in eurythermic fish after cold acclimatization [46].

Kappel et al. (2000) tested whether gangliosides alter channel protein function in a temperature-dependent manner (5–40 °C) using lipid bilayers with alamethicin. They found that bilayers with gangliosides, unlike controls, showed specific maxima in ion channel conductance and stable lifetimes, indicating that gangliosides influence bilayer viscosity and may induce phase transitions, maintaining membrane homeoviscosity around ion channels. This highlights their role in neuronal adaptation to temperature changes [47]. Similar changes were observed in rainbow trout hepatocytes’ lipid rafts [48], though the effects on other sphingomyelins remain unexamined. van Echten and Sandhoff (1989) [49] revealed that thermal adaptation in ^14^C-galactose-labeled murine cerebellar cells leads to significant changes in ganglioside incorporation, with lower temperatures inhibiting *a*-series ganglioside biosynthesis, emphasizing the role of gangliosides in neuronal cell adaptation to different temperatures.

Despite several studies over the past forty years on ganglioside composition in fish, research on their immunohistochemical localization during temperature acclimatization is lacking. For instance, while Avrova (1985) [50] and Hilbig and Rahmann (1980) [51] reported significant findings on ganglioside composition, they did not address localization within brain regions. Viljetić et al. (2012) [52] explored ganglioside localization in Actinopterygian fish brains, but not in relation to temperature changes. This study aims to fill this gap by investigating the seasonal expression and distribution of brain gangliosides in rainbow trout and common carp, contributing to fish neurobiology and ecological physiology.

This study aimed to determine the expression and distribution of gangliosides in specific regions of the rainbow trout and common carp brains regarding seasonal temperature changes. By comparing two fish species with markedly different ecological valences, this research explores how these species regulate ganglioside expression and distribution in response to seasonal temperature fluctuations. The common carp (*Cyprinus carpio* L.), a eurythermic species with a broad tolerance for temperature fluctuations [53], may exhibit more plasticity in ganglioside composition, indicating a flexible biomembrane structure that adapts to diverse environmental conditions. In contrast, the rainbow trout (*Oncorhynchus mykiss* Walbaum), a stenothermic species, thrives in a more restricted thermal range [54], and its limited ganglioside variability suggests a more specialized adaptive response. Ganglioside expression was determined through immunohistochemical analysis in different brain regions, while the types of gangliosides were identified using thin-layer chromatography over a six-month period, with data presented for January, April, and June. January represents the winter period, characterized by lower water temperatures and higher oxygen levels. April, as the spring period, serves as a transition phase, while June reflects the summer regime, with higher water temperatures and lower oxygen concentrations compared to January. This study highlights how ganglioside patterns support physiological adaptation to seasonal temperature shifts, contributing to the understanding of their ecological resilience and neurobiological responses. Given the lack of more recent literature, this research may be particularly relevant in explaining vertebrates’ potential for neuronal temperature adaptation.

## 2. Materials and Methods

### 2.1. Experimental Animals, Ethics, and Tissue Collection

Twenty adult specimens of equal age and size from each of the two fish species, rainbow trout (*Oncorhynchus mykiss* Walbaum, Salmonidae) and common carp (*Cyprinus carpio* L., Cyprinidae), were caught using monofilament nets and sacrificed monthly from January (winter) to June (summer), resulting in a total of forty fish per month. To account for natural temperature variations between day and night at the collection site, all samples were consistently collected between 6:00 a.m. and 8:00 a.m. under natural conditions when temperature fluctuations were minimal, ensuring consistent and reliable assessments of ganglioside expression and distribution. Common carp specimens, with a total length range of 32–35 cm, total weight range of 585–617 g, and brain weight range of 1.15–1.38 g, were obtained from the polycultural earth ponds “Grudnjak” (Ribnjacarstvo PP Orahovica, 45.6377800, 18.0447200). Rainbow trout specimens, with a total length range of 19–22.5 cm, total weight range of 95–121 g, and brain weight range of 0.26–0.38 g, were sourced from small-scale monocultural flow-through ponds at “Valis Aurea” (Turnić, Požega 45.37244125786282, 17.705524150318624) owned by the Odvorčić family. All fish were collected in collaboration with the Faculty of Agrobiotechnical Sciences Osijek, Croatia. Both species were raised in consistent environments that reliably provided the necessary conditions for thriving, including appropriate feed, growth, reproduction, and shelter. The natural environmental conditions (soil, water, temperature, pH, light, and turbidity) supported good survival rates and optimal fish growth. The water temperature and oxygen concentration were measured daily (HI 9146, Hanna Instruments, Woonsocket, RI, USA). All analyzed fish were adults, as confirmed by their size and reproductive ability, and were chosen to represent a consistent size and age range, minimizing variability and ensuring reliable comparisons throughout the study. Specimens were selected based on specific criteria, including fish within the required length range and healthy individuals with no visible signs of disease, parasites, or physical injury. The fish were sacrificed immediately after being caught using an overdose of clove oil (*Eugenia caryophyllus* bud oil, CAS Number 84961-50-2, Aromara d.o.o., Šenkovec, Croatia), followed by decapitation and brain dissection. Ten brains from each species were directly frozen at −80 °C, while another ten brains from each species were fixed in 4% buffered paraformaldehyde for 2 days at 4 °C, cryoprotected in 10% buffered sucrose, and frozen in 2-methylbutane. All samples were stored at −80 °C until immunohistochemical analysis. All analyses were conducted in the Laboratory of Neurobiology at the Department of Medical Biology, School of Medicine, Josip Juraj Strossmayer University of Osijek. The original research was performed following guidelines established by the European Council (E.U., 2010/63). The study protocol was approved by the Ethical Committee of the School of Medicine in Osijek (2158/61-02-145/2-06).

### 2.2. Methods

#### 2.2.1. Ganglioside Extraction

Ganglioside extraction was performed according to the modified protocol by Schnaar [55] using freshly frozen brains. Briefly, a 10% aqueous brain homogenate was prepared using a Teflon pestle (Wheaton Science Products, Millville, NJ, USA) in ice-cold water from a directly frozen brain sample stored at −80 °C. Cold, redistilled extraction solvents were added to the sample in a ratio of chloroform/methanol/water of 1:2:0.75 (*v*/*v*), where water represents the aqueous tissue homogenate. Organic solvents, chloroform, and methanol (Sigma-Aldrich, St. Louis, MO, USA) were redistilled before use. After extraction on ice, the samples were centrifuged for 15 min at 3000 rpm at 4 °C (Centrifuge 5804R, Eppendorf, Berzdorf, Germany). The clear supernatant was separated, and the precipitate was re-extracted at room temperature with double the solvent volume. The re-extracted samples were centrifuged again, and the supernatants were pooled. Gangliosides were separated by phase partitioning, with the upper hydrophilic phase containing the gangliosides. The required water volume for partitioning was 0.173 times the combined supernatants. After adding water, the extract was centrifuged for 15 min at 3000 rpm at room temperature. The upper phases were collected into glass tubes and evaporated in a nitrogen stream at 45 °C (Techne Sample Concentrator, Thermo Fisher Scientific, Göteborg, Sweden). The samples were then dissolved in 1.5 mL of distilled water and purified by dialysis (Spectra/Por Dialysis Membrane Tubing 2) for 72 h, with daily water changes. The dialyzed sample was collected and evaporated under a nitrogen stream at 45 °C (Sample Concentrator, Techne). The extracted gangliosides were dissolved in a chloroform/methanol/water solvent in a ratio of 2:43:55 (volume corresponding to the brain mass) and applied using a Hamilton syringe (7 µg of ganglioside) onto activated thin-layer chromatography plates (HPTLC plates, with a layer of silica gel 60 on glass, Aldrich, Milwaukee, WI, USA; activated by heating at 110 °C for 20 min and cooled), which were then dried and reactivated. The samples, along with a standard containing four complex gangliosides (GM1, GD1a, GD1b, and GT1b), were applied to HPTLC plates. Afterward, the gangliosides were developed in a solvent system for separation consisting of chloroform/methanol/0.25% KCl/2.5 M NH_4_OH (Sigma-Aldrich, St. Louis, MO, USA) in the ratio 60:42:10.5:0.5. The gangliosides were visualized with Svennerholm’s reagent [56]. The plates were scanned, and isolated gangliosides were analyzed using Image J 1.54f software [57]. The results were expressed as percentages of each ganglioside fraction relative to the total ganglioside amount in the sample, as previously described [58].

#### 2.2.2. Immunohistochemical Analysis

The regional expression and variability of brain gangliosides in the two observed fish species were analyzed using free-floating immunohistochemistry on fixed brains. Brain sections (35 µm) were prepared using a cryostat (Leica, CM3050S, Nussloch, Germany) and captured in 24-well dishes in 1×PBS. First, sections were treated with 1% hydrogen peroxide (Kemika, Zagreb, Croatia) for 30 min to eliminate endogenous peroxidase activity. After that, nonspecific binding was blocked with 1% bovine serum albumin (Sigma-Aldrich, St. Louis, MO, USA) and 5% goat serum (Gibco, Invitrogen, Auckland, New Zealand) in 0.1 M PBS for 2 h at 4 °C with continuous shaking (Heidolph Rotamax 120, Schwabach, Germany). Highly specific IgG monoclonal antibodies against gangliosides GM1, GD1a, GD1b, and GT1b (Seikagaku Corporation, Tokyo, Japan) were used. The primary antibodies (anti-GM1 (1:1000), anti-GD1a (1:2000), anti-GD1b (1:2000), and anti-GT1b (1:10,000)), prepared in blocking solution, were incubated with the sections overnight at 4 °C with shaking. The sections were washed three times in cold 0.1 M PBS and incubated with a biotinylated goat anti-mouse IgG or IgM secondary antibody (1:500, Jackson Immunoresearch lab., West Grove, PA, USA) for 4 h at 4 °C with shaking, then washed three times for 10 min, and incubated in the Vector Elite kit tertiary complex (Vector Laboratories, Burlingame, CA, USA) for 2 h at 4 °C with shaking. Visualization was performed using 3,3′-diaminobenzidine (DAB, Peroxidase Substrate System, Vector Lab, Burlingame, CA, USA), resulting in brown to gray coloration where the primary antibody has bound. A negative control was conducted using the same protocol, omitting the primary antibody. The sections were washed in 0.1M PBS, mounted on silanized glass slides, dried, and scanned (Super Coolscan 9000, Nikon, Tokyo, Japan). The sections were covered with Vectamount coverslips (Vector Lab, Burlingame, CA, USA) and analyzed under a microscope (Axioskop 2 MOT, Carl Zeiss, Jena, Germany) with imaging (Olympus DP70, Optical Olympus, Tokyo, Japan). Ganglioside expression was analyzed using Image J 1.54f software [57].

#### 2.2.3. Statistical Analysis

The normality of the data distribution was assessed using the Shapiro–Wilk test. Since the data did not follow a normal distribution, non-parametric statistics were used for all analyses. Although the research was conducted throughout the whole six-month study period, we chose to present data only from January, April, and June, as these months exhibited statistically significant results representing seasonal (thermal) variations. Differences in ganglioside expression across specific brain regions for each fish during these months were determined by the Kruskal–Wallis test with post hoc Dunn’s test and Bonferroni correction. The same tests were used to analyze differences in the proportion of each ganglioside in the total composition of gangliosides for each month. All analyses were conducted at a significance level of alpha 0.05 using the statistical package SPSS.

## 3. Results

**Figure 1:**
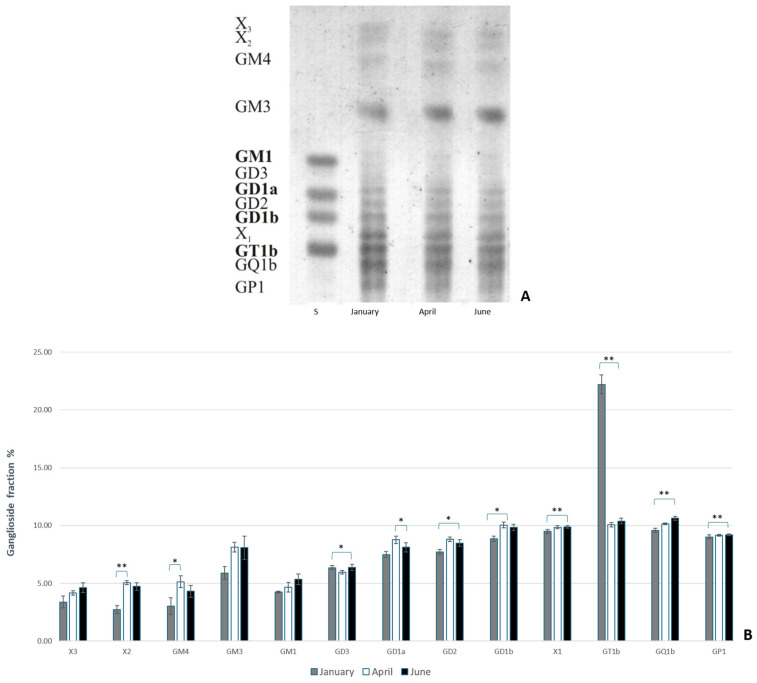
(**A**) The thin-layer chromatography of gangliosides extracted from rainbow trout brains from January, April, and June. (**B**) The percentage of each ganglioside fraction relative to all extracted gangliosides in the brains of rainbow trout. Abbreviations: X_1_, X_2_, and X_3_ = unknown gangliosides; S = standard. Images were processed with Biorender [59]. Significantly different results are indicated (* *p* ˂ 0.05; ** *p* ˂ 0.01 pairwise comparison).

**Figure 2:**
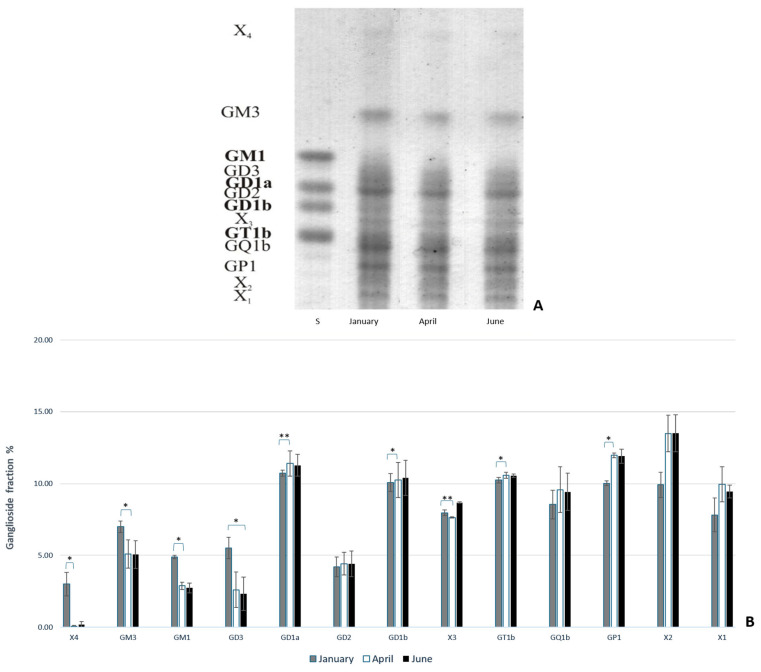
(**A**) The thin-layer chromatography of gangliosides extracted from common carp brains from January, April, and June. (**B**) The percentage of each ganglioside fraction relative to all extracted gangliosides in the brains of common carp. Abbreviations: X_1_, X_2_, X_3_, and X_4_ = unknown gangliosides; S = standard. Images were processed with Biorender [59]. Significantly different results are indicated (* *p* ˂ 0.05; ** *p* ˂ 0.01 pairwise comparison).

To analyze the ganglioside composition in the brains of common carp and rainbow trout from winter (January) to summer (June), thin-layer chromatography (TLC) was performed. This analysis provided insight into the different types of expressed gangliosides and how they change in response to thermal (heat) adaptation. The results indicate that polysialogangliosides are dominantly expressed in the brains of both species (Figure 1 and Figure 2). Gangliosides in the brains of both species are expressed as a percentage of each ganglioside fraction relative to the total gangliosides, with significant changes observed over a six-month period. The results also reveal species-specific differences in the ganglioside fraction percentage in response to heat adaptation. During acclimatization to higher temperatures, an increase in polysialylated ganglioside fractions was observed in both common carp and rainbow trout (Figure 1B and Figure 2B). At lower water temperatures, a shift in ganglioside biosynthetic pathways from the *a*- to the *b*-pathway was noted.

In rainbow trout, the expression of simple gangliosides (X_2_ and GM4) increased in April (*p* = 0.005 for X_2_ and *p* = 0.032 for GM4), with no significant changes in June compared to January, while the expression of most polysialogangliosides (GD3, GD2, GD1b, GT1b, GQ1b, and GP1) increased in June compared to January (*p* = 0.013 for GD3; *p* = 0.024 for GD2 and GD1b; *p* = 0.007 for GT1b; and *p* = 0.005 for GQ1b and GP1) and compared to April, with only GD1a showing a significant increase (*p* = 0.024) (Figure 1B).

In common carp, the expression of GD3 significantly decreased in June compared to January (*p* = 0.032). Additionally, the percentages of X_4_, GM3, and GM1 gangliosides significantly decreased in April (*p* = 0.023 for X_4_; *p* = 0.024 for GM3; and *p* = 0.043 for GM1) compared to January (Figure 2B).

The measured water temperature values and concentration of dissolved oxygen are presented in the Supplementary Materials in Tables S1 and S2.

**Figure 3:**
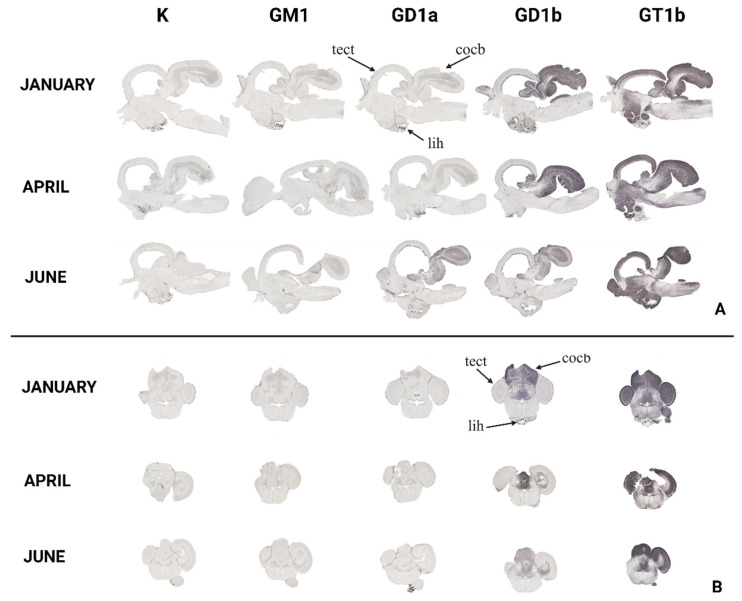
The expression of complex gangliosides (GM1, GD1a, GD1b, and GT1b) in the brains of rainbow trout on sagittal (**A**) and coronal (**B**) sections in different seasons (January—winter; April—spring; and June—summer). Abbreviations: K—negative control; tel—*telencephalon*; tect—*tectum opticum*; lih—*lobus inferior hypothalami*; cor—*corpus cerebelli*; crcb—*crista cerebellaris*.

**Figure 4:**
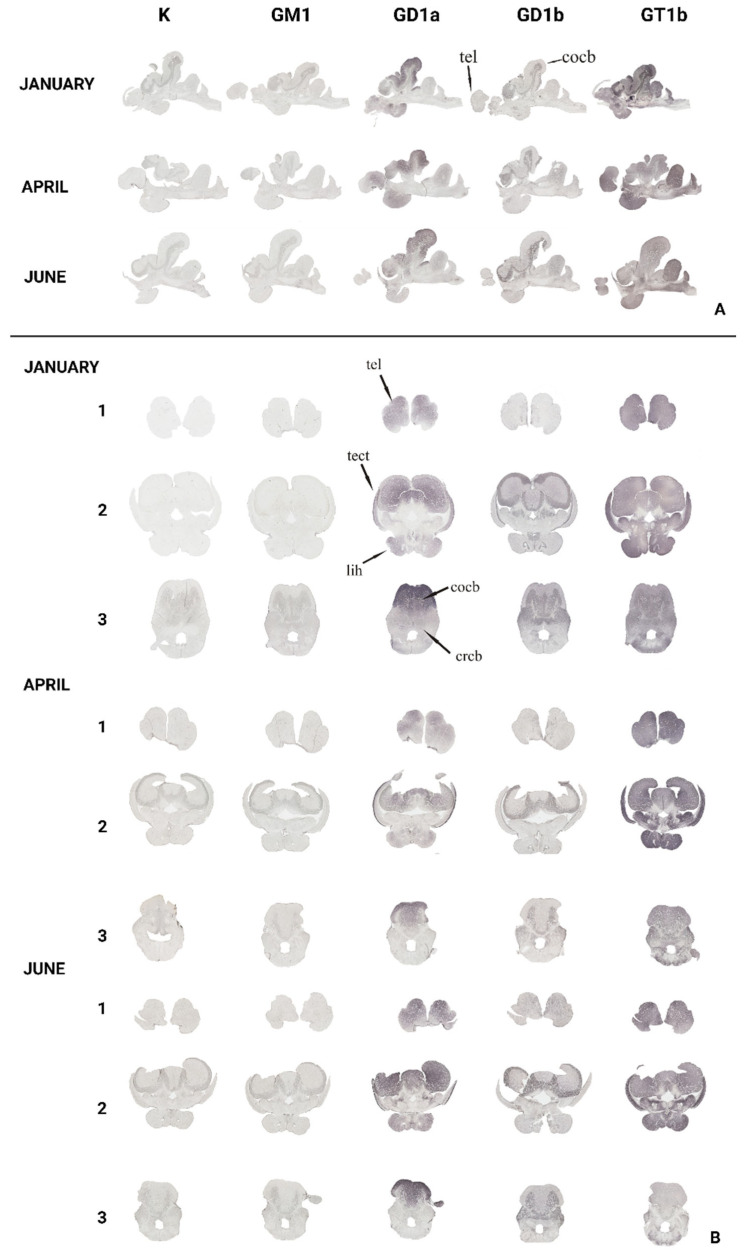
The expression of complex gangliosides (GM1, GD1a, GD1b, and GT1b) in the brains of common carp on sagittal (**A**) and coronal (**B**) sections in different seasons (January—winter; April—spring; and June—summer). Abbreviations: K—negative control; tel—*telencephalon*; tect—*tectum opticum*; cor—*corpus cerebelli*.

To gain insight and a deeper understanding of ganglioside function related to thermal (heat) adaptation, their expression and distribution were analyzed in functionally different brain regions: *corpus cerebelli*, *crista cerebelli*, *lobus inferior hypothalamic*, and *tectum* opticum in rainbow trout (Figure 3) and common carp brains (Figure 4). The results confirmed the dominance of polysialogangliosides in the brains of both fish species. Additionally, ganglioside expression was region-specific and related to thermal (heat) adaptation.

**Figure 5:**
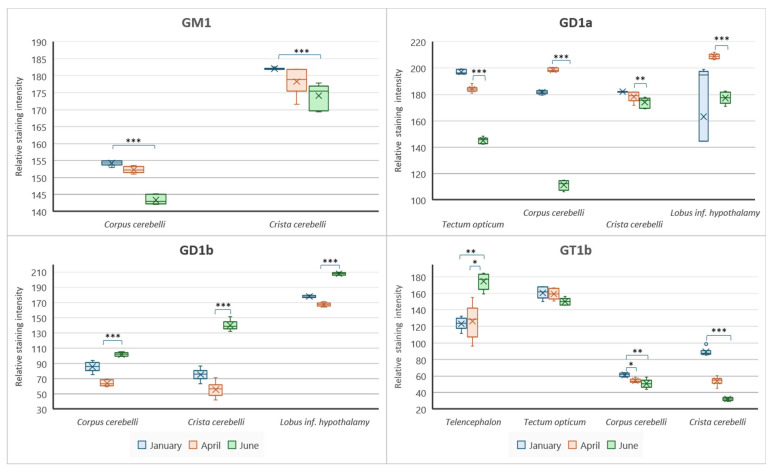
Graphic presentation of changes in brain ganglioside expression in rainbow trout brain regions. Data are presented as medians with upper and lower distribution limits, with *n* = 18 independent determinations per data point. Significantly different results are indicated (* *p* ˂ 0.05; ** *p* ˂ 0.01; *** *p* ˂ 0.001 pairwise comparison). Relative staining intensity: 0 = greatest intensity; 255 = no staining.

**Figure 6:**
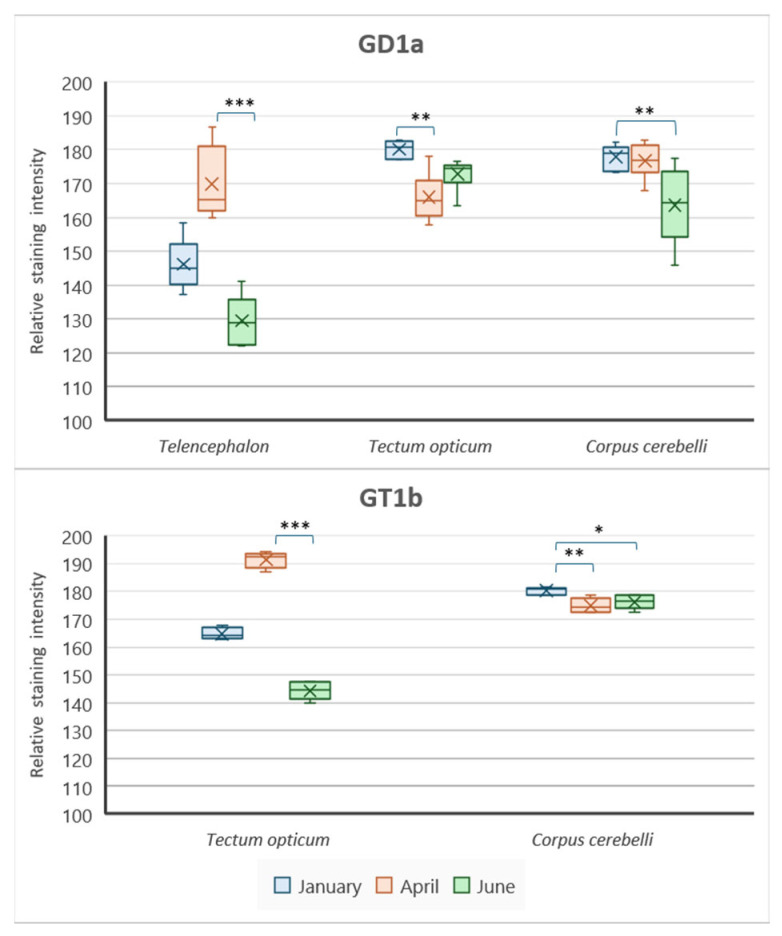
Graphic presentation of changes in brain ganglioside expression in common carp brain regions. Data are presented as medians with upper and lower distribution limits, with *n* = 18 independent determinations per data point. Significantly different results are indicated (* *p* ˂ 0.05; ** *p* ˂ 0.01; *** *p* ˂ 0.001 pairwise comparison). Relative staining intensity: 0 = greatest intensity; 255 = no staining.

Major brain gangliosides were analyzed in functionally specific brain regions to connect their role with the thermal adaptation process. In addition to differences in distribution across specific brain regions, both rainbow trout (Figure 5) and common carp (Figure 6) exhibited quantitative differences in the expression of major brain gangliosides.

### 3.1. The Forebrain—Telencephalon

**Figure 7:**
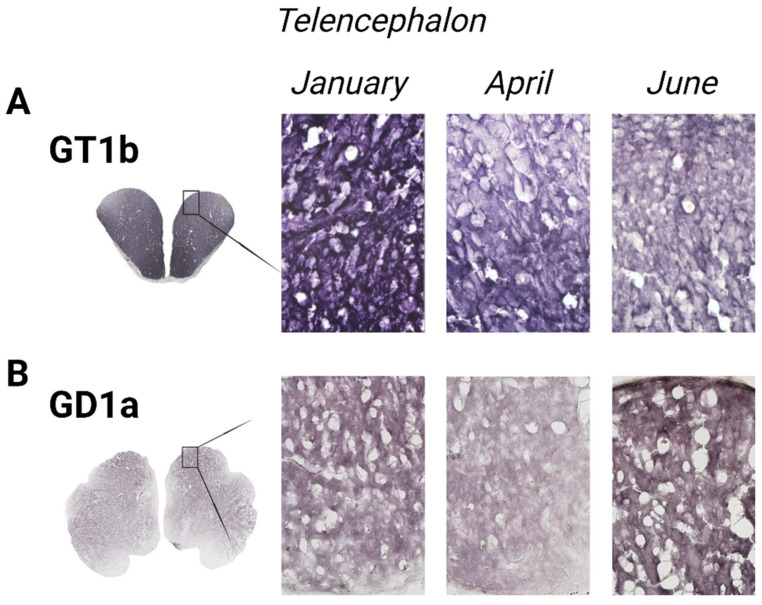
Immunohistochemical expression of gangliosides in the telencephalon of rainbow trout (**A**) and common carp (**B**) in different seasons (January—winter; April—spring; and June—summer). Scanned brain sections (**left**), with detailed insight under 200× magnification (**right**).

The variation in GT1b ganglioside expression in the *telencephalon* of rainbow trout and GD1a expression in common carp changed across the analyzed months (Figure 7). In the rainbow trout brain, GT1b expression significantly decreased in June (*p* = 0.006) compared to January (Figure 5). Conversely, GD1a expression in the telencephalon of common carp significantly increased in June (*p* < 0.001) compared to April (Figure 6).

### 3.2. The Optic Tectum—Tectum Opticum

**Figure 8:**
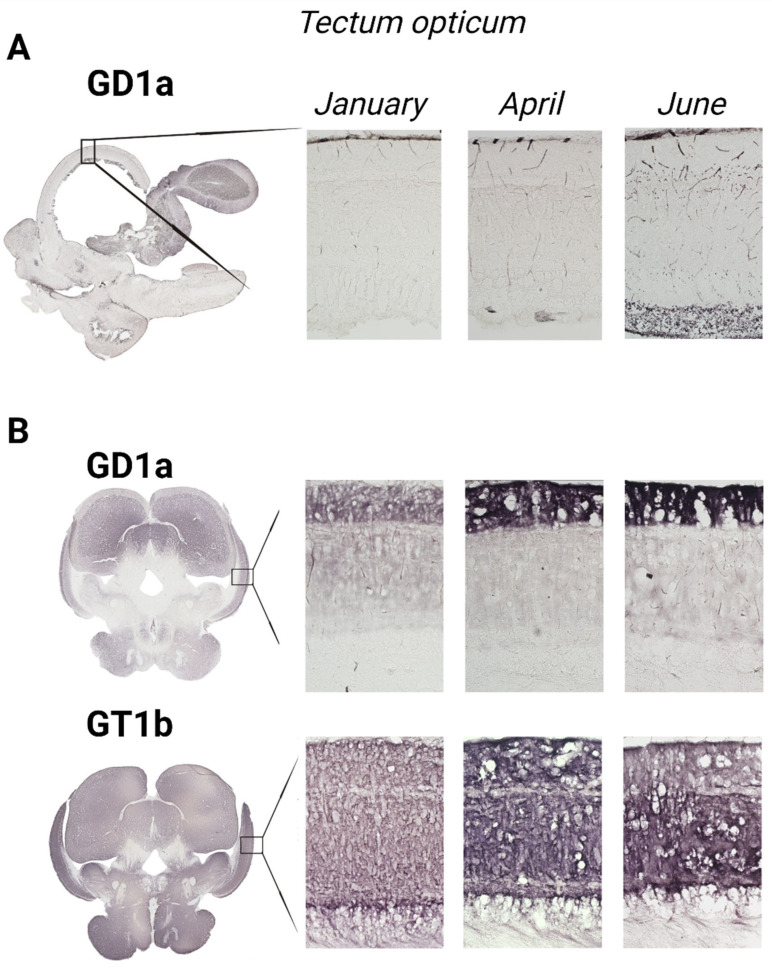
Immunohistochemical expression of gangliosides in the tectum opticum of rainbow trout (**A**) and common carp (**B**) in different seasons (January—winter; April—spring; and June—summer). Scanned brain sections (**left**), with detailed insight under 100× magnification (**right**).

According to Figure 8, both rainbow trout (*p* = 0.001) and common carp (*p* = 0.004) exhibited a significant increase in GD1a expression in the *tectum opticum*, while GT1b expression significantly increased only in the common carp brain (*p* = 0.001) (Figure 5 and Figure 6).

### 3.3. The Cerebellar Crest—Crista cerebelli

**Figure 9:**
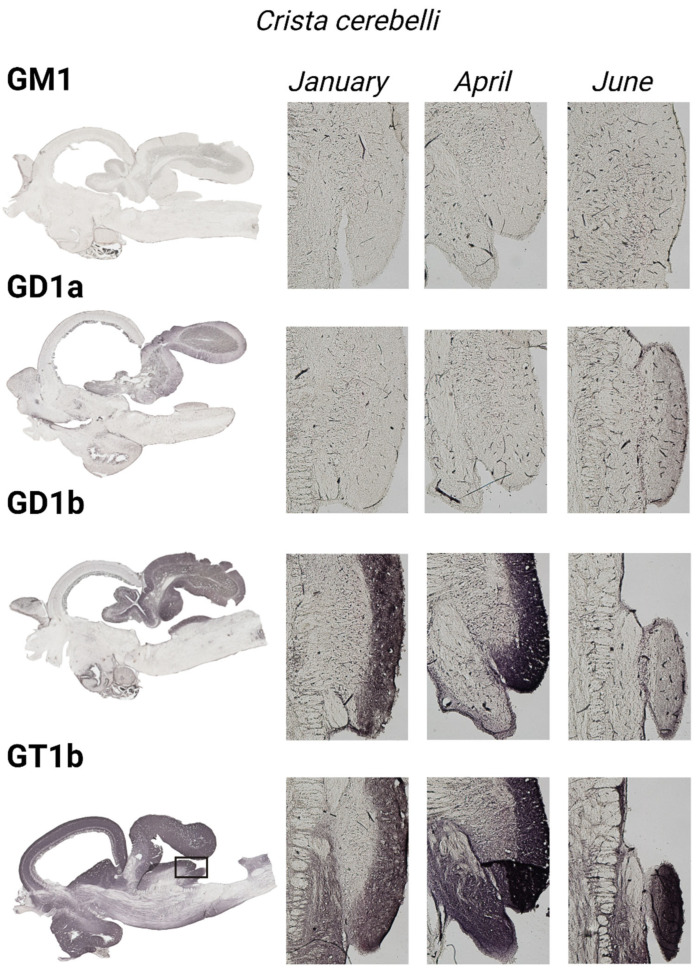
Immunohistochemical expression of gangliosides in the crista cerebelli of rainbow trout in different seasons (January—winter; April—spring; and June—summer). Scanned brain sections (**left**), with detailed insight under 50× magnification (**right**).

All four major brain gangliosides were expressed in the *crista cerebelli* of rainbow trout (Figure 9), but no positive reaction was observed in common carp. The expression of GM1 (*p* = 0.001), GD1a (*p* = 0.002), and GT1b (*p* < 0.001) significantly increased in June compared to January, while GD1b expression decreased (*p* < 0.001) (Figure 5).

### 3.4. The Cerebellar Corpus—Corpus cerebelli

**Figure 10:**
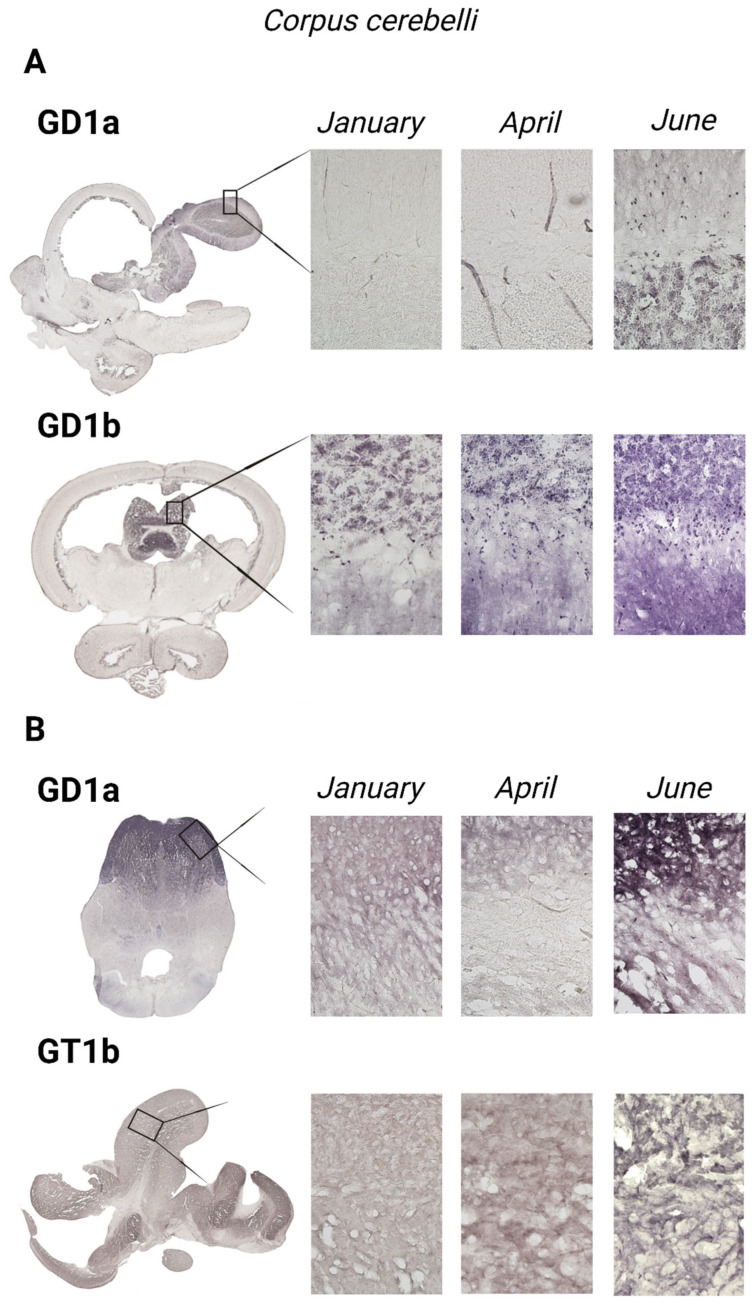
Immunohistochemical expression of gangliosides in the *corpus cerebelli* of rainbow trout (**A**) and common carp (**B**) in different seasons (January—winter; April—spring; and June—summer). Scanned brain sections (**left**), with detailed insight under 200× magnification (**right**).

All four major brain gangliosides were expressed in the *corpus cerebelli* of rainbow trout, whereas only GD1a and GT1b showed a positive reaction in the common carp brain (Figure 10). In rainbow trout, the expression of GM1 (*p* < 0.001), GD1a (*p* < 0.001), and GT1b (*p* = 0.006) significantly increased in June compared to January, while GD1b expression decreased (*p* < 0.001) (Figure 5), mirroring the pattern observed in the *crista cerebelli*. In the *corpus cerebelli* of common carp, GD1a (*p* = 0.033) and GT1b (*p* = 0.028) expression also significantly increased in June (Figure 5 and Figure 6).

### 3.5. The Inferior Lobe of the Hypothalamus—Lobus inferior hypothalami

**Figure 11:**
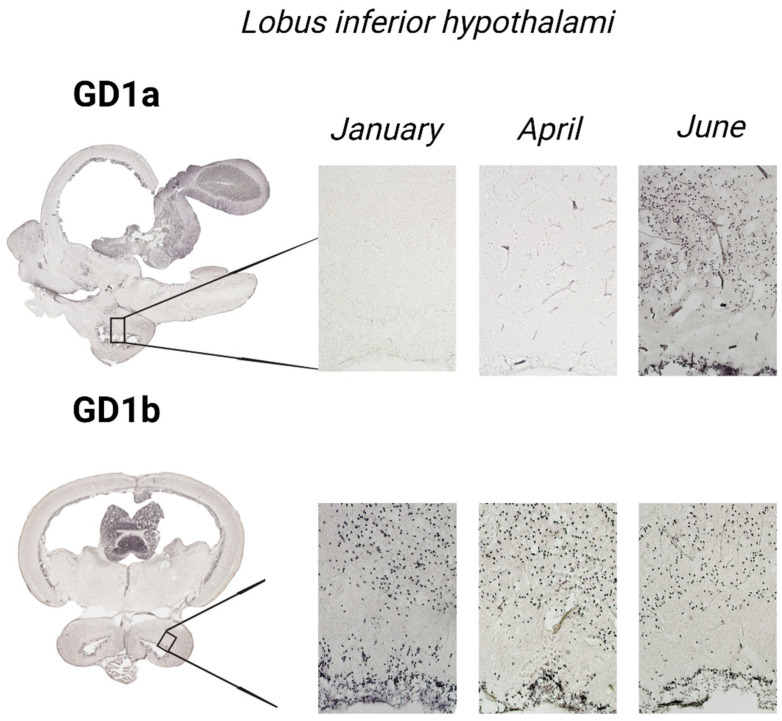
Immunohistochemical expression of ganglioside in the *lobus inferior hypothalami* of rainbow trout in different seasons (January—winter; April—spring; and June—summer). Scanned brain sections (**left**), with detailed insight under 100× magnification (**right**).

In rainbow trout (Figure 11), a significant increase in GD1a expression (*p* = 0.001) was detected in June, while GD1b expression decreased (*p* < 0.001). No significant changes were observed in the common carp brain.

## 4. Discussion

In this work, changes in ganglioside expression were observed for the first time in the brains of two fish species under natural environmental conditions, where fish are subject to complex changes. As conditions fluctuate over time, fish can remain within a suitable range by moving between different parts of their habitat [60]. The temperature in certain areas of the lake fluctuates throughout the day, allowing fish to choose the optimal temperature throughout the day by actively moving [61]. Long-term adverse environmental conditions, such as low water temperature, a lack of nutritious organisms, diseases caused by physico-chemical parameters, and infectious and invasive diseases, can lead to a decrease in overall growth intensity [62]. The results indicate species-specific differences in the percentage of ganglioside fractions as a response to heat adaptation.

The rainbow trout (*Oncorhynchus mykiss*) and common carp (*Cyprinus carpio*) are both freshwater fish but differ significantly in their biology, ecology, behavior, and ecological preferences. Rainbow trout prefer cold, well-oxygenated waters, typically found in streams, rivers, and cold-water lakes. They thrive in temperatures ranging from 10 to 16 °C (50 to 60 °F) and are sensitive to warmer temperatures [54]. In contrast, common carp are more tolerant of a wide range of environmental conditions, including warmer and more turbid waters. They can survive in temperatures from 3 to 35 °C (37 to 95 °F) and can adapt to low-oxygen conditions, making them more versatile in different habitats [53,63]. As a eurythermic species, common carp exhibited smaller changes in ganglioside composition and distribution compared to rainbow trout, which are stenothermic species [64]. This aligns with the observation that even mammals, as well-adapted species, exhibit a uniform distribution of gangliosides [44].

The differing expression of gangliosides in winter compared to summer supports the studies indicating that high-temperature stress leads to physiological changes in the fish brain, resulting in transcriptional fluctuations [65,66,67,68]. Additionally, the distinct distribution and specific composition of gangliosides in certain brain regions strongly suggest that they have important and specific functions in these regions. The results of this study could help explain the subsequent transformation of temperature sensation into thermoregulatory behaviors in fish [69]. The observed increased expression of polysialylated ganglioside fractions in the brains of both common carp and rainbow trout suggests that, in response to higher temperatures, their brain cells might adapt by modifying membrane composition, particularly by increasing the amounts of these more complex gangliosides. This adaption may serve as a protective mechanism to maintain membrane stability or alter cell signaling pathways for better function in a warmer environment. Other studies have also indicated that adaptation to environmental temperature is influenced more by the presence of certain polysialogangliosides than by the total ganglioside content [28,70], suggesting that ganglioside function is closely related to thermal conditions [71,72].

Interestingly, common carp showed noticeable changes in ganglioside expression in April, while rainbow trout exhibited significant changes later, in June. The speed of adaptation to temperature changes is limited by the rate of changes in ganglioside metabolism [70]. Consequently, organisms with different ganglioside metabolisms exhibit varying sensitivities to temperature changes and adaptation speeds [73]. While fish can activate sensorimotor pathways for reflex escape within 30 s in response to a sudden 10 °C drop in temperature [74] and initiate a whole-body stress response within 90 s by reducing blood volume and redirecting blood flow from the brain to avoid rapid cooling [75], natural adaptation from 20 °C to 4 °C typically takes 4–6 weeks [51]. This slow neurobiological adaptation does not align with the 2-week change in phospholipid and cholesterol composition but matches the 4–6-week change in ganglioside composition. During the thermal acclimatization of fish, the polarity of gangliosides in neuronal membranes changed, while alterations in other lipids were recorded in the liver and muscles [33]. Rainbow trout exhibited increased expression of simple gangliosides (X_2_ and GM4) early in the year (April), followed by a significant rise in complex gangliosides (polysialogangliosides) as temperatures continued to increase into June. This suggests a phased adaptation, where initial simple adjustments are followed by more complex biochemical changes, indicating that the brain is preparing for warmer conditions, as April marks the transition from winter to spring. Conversely, common carp displayed a different pattern, with a significant decrease in a specific polysialoganglioside (GD3) in June and in several gangliosides (X_4_, GM3, GM1) in April. This could indicate that common carp have a different or more streamlined strategy for adjusting to temperature changes, making them less sensitive to heat stress than rainbow trout, which aligns with their ecological temperature valence.

The opposite pattern of GD3 expression in June (decreasing in common carp and increasing in rainbow trout) could be explained by previous studies on mammals, which indicate that GD3 plays a key role in apoptosis by initiating the opening of the mitochondrial permeability transition pore [76]. Its neuroprotective effects may stem from a reduction in GD3-induced apoptosis [77]. These differences between the species highlight how distinct environmental adaptations are reflected at the molecular level, particularly in the brain ganglioside composition during seasonal temperature fluctuations.

The results of this study indicate that ganglioside expression is region-specific and related to seasonal heat adaptation, meaning that the expression of gangliosides varies across different areas or regions of the brain. In other words, certain regions of the body may have higher or lower levels of gangliosides, depending on local conditions or functions, suggesting that gangliosides play a role in helping the body adapt to seasonal changes in temperature, particularly heat. The different distributions and specific compositions of gangliosides in certain regions of the brain strongly suggest that they have important and specific functions in these areas, and the results of this study could explain the subsequent transformation of temperature sensation into thermoregulatory behaviors in fish [69]. Gangliosides are crucial in modulating signals from leptin receptors and potentially various other receptors involved in energy homeostasis, in a manner specific to each ganglioside species [78]. During acute cold stress in ectotherms, lactate utilization and transport pathways are essential for maintaining energy homeostasis and vital brain cell functions [79]. Lactate helps sustain essential brain cell activities under stress by providing an alternative energy source and can impact synaptic activity and plasticity [80] as well as neuronal excitability, which are critical for maintaining proper brain function. Studies have shown that lactate levels can affect neuronal activity and synaptic transmission in various brain regions, including the fish brain [24,81]. This is particularly relevant for the *lobus inferior hypothalamic* [82], the largest laterally located brain region involved in integrating visual and gustatory signals for food searching. This region receives input from visual centers in the pretectum [83] and secondary gustatory centers [84] and is crucial for regulating stress and maintaining homeostasis. The telencephalon, which processes sensory information and coordinates behavioral responses related to temperature changes, supports thermal adaptation [14,85]. Gangliosides, which are essential for maintaining these processes, also become particularly important under thermal stress. This study showed that GT1b levels significantly decreased in the *telencephalon* and *lobus inferior hypothalami* of rainbow trout, indicating a shift in ganglioside composition in response to thermal adaptation. In contrast, GD1a expression significantly increased in common carp, highlighting a different adaptive response. This biochemical shift suggests a possible interrelationship between lactate utilization and ganglioside modulation in managing stress and maintaining function in the brain under temperature fluctuations.

In this study, all four major brain gangliosides were expressed in the *corpus cerebelli* of rainbow trout, while only GD1a and GT1b were present in the carp brain. Compared to January, the levels of these gangliosides significantly increased in June in both fish species. Similar trends were observed in the *crista cerebelli*. The integration of eye and head movements with body movements is one of the basic movements controlled by the cerebellum, which is vital for fish like rainbow trout that use sight to search for food [86]. Previous research has indicated that sensitivity to heat is mediated by the cerebellum [87]. Cold receptors differ from heat receptors in that they respond to changes in temperature rather than to absolute temperature.

The *lobus inferior hypothalami* and *telencephalon* are crucial for integrating sensory inputs and regulating stress and homeostasis in fish, particularly under thermal stress. Rainbow trout, which inhabit colder environments, showed a decrease in GT1b, a ganglioside associated with membrane stability and found in mature neurons [88,89]. This reduction might help optimize membrane fluidity and neuronal function, enhancing adaptation to lower temperatures. In contrast, common carp, with broader temperature tolerance, exhibit an increase in GD1a, a ganglioside involved in synaptic plasticity and a marker of synaptogenesis [88,89]. This increase may improve neuronal adaptability and functional plasticity, aiding the carp’s ability to manage temperature fluctuations. These differences in ganglioside expression underscore their role in thermal adaptation, reflecting the respective environmental niches and physiological requirements of each species. Seasonal variations in ganglioside levels in the cerebellum and other brain regions suggest that these adjustments are part of the fish’s broader strategy to manage temperature fluctuations. The cerebellum’s role in coordinating movements and its sensitivity to temperature further supports the need for effective thermal adaptation mechanisms.

Changes in water temperature can significantly impact the concentration of dissolved oxygen, which, in turn, affects the ganglioside distribution in fish brains. As temperatures rise during summer, oxygen levels decrease, potentially leading to a decrease in complex gangliosides such as GT1b. This decrease could be an adaptive response to thermal stress, reflecting how fish adjust their neural chemistry to maintain functionality under changing conditions. Previous studies have demonstrated that hypoxia can lead to decreases in brain gangliosides [90]. Considering that these species have distinct ecological valency with respect to temperature and oxygen content, distinct seasonal variations in gangliosides across different brain regions were anticipated. Understanding these adaptations is crucial, especially given the ecological and economic significance of species like rainbow trout and common carp. Rainbow trout, important predators in their ecosystems, can influence the population dynamics of other species, while common carp, known as an invasive species in several regions [91,92,93], can alter aquatic ecosystems by modifying sediment composition and outcompeting native species. By studying how these species adapt to seasonal temperature and oxygen fluctuations, we gain insights into their behavioral and physiological resilience. This knowledge is vital for developing effective conservation strategies and managing their ecological impacts, ensuring the protection of critical habitats and the stability of aquatic ecosystems.

### Limitations of the Study and Future Research Directions

While this study offers valuable insights into the role of gangliosides in thermal adaptation, several limitations should be noted. The focus on two fish species, though useful, limits the generalizability of the findings, and the study’s specific seasonal and environmental context may restrict its applicability to other settings. Although this study primarily focused on temperature variations, it was conducted under natural conditions, where factors such as photoperiod were observed with their natural fluctuations rather than being controlled. This approach reflects typical environmental conditions and provides a comprehensive understanding of how temperature interacts with other environmental variables in influencing ganglioside expression and distribution. Future studies could explore how the combined effects of temperature and photoperiod interact over longer periods to influence the sensory and circadian rhythms of fish, potentially improving strategies for managing environmental stressors in aquaculture. Additionally, the precise molecular mechanisms driving the observed changes were not explored.

Further research should focus on the interplay between lipid metabolism and ganglioside composition, particularly regarding brain area function and endocrine regulation. Lipid metabolism is essential for maintaining the structure and function of cell membranes, especially in neurons, where gangliosides are abundant [94]. Ceramides, synthesized from serine and palmitoyl-CoA in the endoplasmic reticulum, serve as precursors for ganglioside synthesis and are transported to the Golgi for further processing by glycosyltransferases, which generate cell-type-specific glycolipid patterns [95]. During stress, the activity of these enzymes may be altered, leading to shifts in ganglioside profiles that help maintain cellular stability under fluctuating temperatures [96,97]. For example, increased glycosyltransferase activity during stress [98] can enhance the synthesis of polysialylated gangliosides, which are important for neuroplasticity [99,100]. Moreover, glycosyltransferases rely on lipid metabolism for substrate availability [101], and shifts in lipid metabolism can modulate the expression or activity of these enzymes, resulting in changes in ganglioside profiles across different brain regions [102]. Investigating the expression levels of these enzymes under different temperature conditions would provide valuable insights into the specific molecular mechanisms and biochemical pathways through which gangliosides contribute to thermal adaptation. Functional studies using enzyme inhibitors could further explain the impact of temperature on ganglioside metabolism. Additionally, exploring how gangliosides interact with membrane proteins and other lipids to stabilize cell membranes can help identify key regulatory factors involved in thermal adaptation. Changes in dietary fatty acid composition can significantly impact membrane lipid profiles. According to Roy et al. (2020), trout fed diets rich in eicosapentaenoic acid (EPA) and docosahexaenoic acid (DHA) exhibited increased brain levels of both enzymatic and non-enzymatic metabolites, contributing to anti-inflammatory and neuroprotective effects [103]. Therefore, it is essential to investigate how variations in dietary lipid intake influence ganglioside profiles across different brain regions in fish. Understanding how different lipid sources affect ganglioside synthesis and distribution could reveal important insights into the role of dietary fats in neuronal health and function.

The adaptation of gangliosides over a 4–6-week period [51] underlines that long-term studies are not always required to observe significant metabolic shifts. This study, while short-term, provides valuable insights into how these species adapt to temperature fluctuations, which has implications for both medium- and long-term physiological and ecological dynamics. For rainbow trout, understanding optimal temperature ranges is crucial for preserving their predatory role and maintaining ecosystem balance. Insights into the common carp’s thermal adaptation assist in controlling its invasive species impact, reducing competition and ecological disruptions. Identifying temperature thresholds for physiological performance enables more effective water temperature management, benefiting both natural and aquaculture settings. This knowledge supports conservation strategies by protecting critical habitats for rainbow trout and controlling common carp spread in sensitive areas. In the medium and long term, these findings can significantly enhance aquaculture practices. Ultimately, optimizing water temperatures can enhance aquaculture practices, improving growth, health, and reproductive success for both species and supporting sustainable production and conservation efforts. Despite these limitations, this study provides valuable insights into how gangliosides contribute to thermal adaptation in fish. It has potential implications for fish health, aquaculture, and conservation, offering a basis for developing strategies to enhance thermal resilience in aquaculture species and protect vulnerable wild populations. Although this study primarily contributes to the fundamental understanding of fish neurobiology, the knowledge acquired can be applied to develop practical strategies for temperature management in aquaculture systems, ultimately supporting better management practices and improving the well-being of cultured fish.

## 5. Conclusions

This study provides significant insights into the role of temperature as a crucial environmental factor influencing fish adaptation through the modulation of brain ganglioside composition. The results reveal distinct patterns of ganglioside variation in response to seasonal temperature fluctuations in both eurythermic common carp and stenothermic rainbow trout. This underscores the importance of gangliosides in maintaining neuronal stability and function under varying thermal conditions. The observed increase in polysialylated gangliosides in common carp and trisialogangliosides in rainbow trout suggest species-specific adaptive mechanisms that contribute to their survival in different thermal environments. These modifications likely help maintain membrane fluidity and stability, essential for effective signal transduction and overall neuronal function during temperature changes. Furthermore, the differential immunohistochemical localization of gangliosides across various brain regions implies that these molecules may have specific functional roles in different parts of the brain, which are crucial for coping with environmental stressors. As climate-induced temperature fluctuations become more frequent and intense, understanding thermal adaptation mechanisms in fish is crucial. This research lays the groundwork for further studies on how ganglioside composition might be manipulated to enhance the resilience of fish and potentially other animals to changing environmental conditions. Such insights could inform conservation strategies and aquaculture practices aimed at mitigating the impacts of climate change. These findings also have broader implications for understanding how other poikilothermic organisms might adapt to environmental stressors, potentially contributing to strategies that enhance species resilience in the face of global climate change.

## Data Availability

The data presented in this study are available on request from the corresponding author.

## Supplementary Materials

The following supporting information can be downloaded at https://www.mdpi.com/article/10.3390/life14101273/s1: Figure S1: The thin-layer chromatography of gangliosides extracted from rainbow trout brains from January to June. Abbreviations: 1 = January, 2 = February, 3 = March, 4 = April, 5 = May, 6 = June, S = standard; Figure S2: The thin-layer chromatography of gangliosides extracted from common carp brains from January to June. Abbreviations: 1 = January, 2 = February, 3 = March, 4 = April, 5 = May, 6 = June, S = standard. Table S1: Measured values of water temperature (Tw) and air (Ta) and dissolved oxygen (O_2_) concentration in water at 9 h during the experimental period for rainbow trout, location Turnić, Požega; Table S2. Measured values of water temperature (Tw) and air (Ta) and dissolved oxygen (O_2_) concentration in water at 9 h during the experimental period for common carp, location Grudnjak, Orahovica.

## References

[1] Priya A.K., Muruganandam M., Rajamanickam S., Sivarethinamohan S., Gaddam M.K.R., Velusamy P., Gamathi R., Ravindiran G., Gurugubelli T.R., Muniasamy S.K. (2023). Impact of Climate Change and Anthropogenic Activities on Aquatic Ecosystem—A Review. Environ. Res..

[2] da Silva J.C., Soares C.M., Bialetzki A. (2024). Effect of an Invasive Fish Species on Nutrient Cycling and on the Community Structure: An Experimental Approach. Aquatic Ecology.

[3] Guo T., Wang Y., Li J., Guo X., Xu S., Han H., Yu J., Li J., Liu Q. (2024). Accumulated CO_2_ Affects Growth, Acid-Base Regulation and Ion Balance of Turbot (*Scophthalmus maximus*) in a Recirculating Aquaculture System. Aquaculture.

[4] Oehlert A.M., Garza J., Nixon S., Frank L., Folkerts E.J., Stieglitz J.D., Lu C., Heuer R.M., Benetti D.D., del Campo J. (2024). Implications of Dietary Carbon Incorporation in Fish Carbonates for the Global Carbon Cycle. Sci. Total Environ..

[5] Dosdat A., Servais F., Métailler R., Huelvan C., Desbruyères E. (1996). Comparison of Nitrogenous Losses in Five Teleost Fish Species. Aquaculture.

[6] de Carvalho D.R., Sparks J.P., Flecker A.S., Alves C.B.M., Moreira M.Z., Pompeu P.S. (2021). Nitrogen Pollution Promotes Changes in the Niche Space of Fish Communities. Oecologia.

[7] Little A.G., Loughland I., Seebacher F. (2020). What Do Warming Waters Mean for Fish Physiology and Fisheries?. J. Fish Biol..

[8] Du J., Xie M., Wang Y., Chen Z., Liu W., Liao J., Chen B. (2020). Connectivity of Fish Assemblages along the Mangrove-Seagrass-Coral Reef Continuum in Wenchang, China. Acta Oceanol. Sin..

[9] Baladrón A., Costa M.J., Bejarano M.D., Pinheiro A., Boavida I. (2021). Can Vegetation Provide Shelter to Cyprinid Species under Hydropeaking?. Sci. Total Environ..

[10] Silveira R., Leão-Neto W.M., Barbosa da Silva F.H. (2020). Small-Sized Fish as Possible Seed Dispersers: Disclosing Novel Fish and Plant Species Interactions in the Pantanal Wetland. Stud. Neotrop. Fauna Environ..

[11] Whitfield A.K., Elliott M. (2002). Fishes as Indicators of Environmental and Ecological Changes within Estuaries: A Review of Progress and Some Suggestions for the Future. J. Fish Biol..

[12] Seebacher F., Narayan E., Rummer J.L., Tomlinson S., Cooke S.J. (2023). How Can Physiology Best Contribute to Wildlife Conservation in a Warming World?. Conserv. Physiol..

[13] Chmura H.E., Williams C.T. (2022). A Cross-Taxonomic Perspective on the Integration of Temperature Cues in Vertebrate Seasonal Neuroendocrine Pathways. Horm. Behav..

[14] Alfonso S., Gesto M., Sadoul B. (2021). Temperature Increase and Its Effects on Fish Stress Physiology in the Context of Global Warming. J. Fish Biol..

[15] Crawshaw L.I. (1977). Physiological and Behavioral Reactions of Fishes to Temperature Change. J. Fish. Res. Board Can..

[16] Islam M.J., Kunzmann A., Slater M.J. (2022). Responses of Aquaculture Fish to Climate Change-Induced Extreme Temperatures: A Review. J. World Aquac. Soc..

[17] Madeira D., Madeira C., Costa P.M., Vinagre C., Pörtner H.-O., Diniz M.S. (2020). Different Sensitivity to Heatwaves across the Life Cycle of Fish Reflects Phenotypic Adaptation to Environmental Niche. Mar. Environ. Res..

[18] Brulé T., Renán X., Colás-Marrufo T. (2022). Potential Impact of Climate Change on Fish Reproductive Phenology: A Case Study in Gonochoric and Hermaphrodite Commercially Important Species from the Southern Gulf of Mexico. Fishes.

[19] Liu H., Yang R., Fu Z., Yu G., Li M., Dai S., Ma Z., Zong H. (2023). Acute Thermal Stress Increased Enzyme Activity and Muscle Energy Distribution of Yellowfin Tuna. PLoS ONE.

[20] Sage R.F. (2020). Global Change Biology: A Primer. Glob. Chang. Biol..

[21] Madaro A., Kristiansen T.S., Pavlidis M.A., Kristiansen T.S., Fernö A., Pavlidis M.A., van de Vis H. (2020). How Fish Cope with Stress?. The Welfare of Fish.

[22] Biederman A.M., Kuhn D.E., O’Brien K.M., Crockett E.L. (2019). Physical, Chemical, and Functional Properties of Neuronal Membranes Vary between Species of Antarctic Notothenioids Differing in Thermal Tolerance. J. Comp. Physiol. B.

[23] Vindas M.A., Fokos S., Pavlidis M., Höglund E., Dionysopoulou S., Ebbesson L.O.E., Papandroulakis N., Dermon C.R. (2018). Early Life Stress Induces Long-Term Changes in Limbic Areas of a Teleost Fish: The Role of Catecholamine Systems in Stress Coping. Sci. Rep..

[24] Gesto M., López-Patiño M.A., Hernández J., Soengas J.L., Míguez J.M. (2013). The Response of Brain Serotonergic and Dopaminergic Systems to an Acute Stressor in Rainbow Trout: A Time Course Study. J. Exp. Biol..

[25] Sandblom E., Clark T.D., Gräns A., Ekström A., Brijs J., Sundström L.F., Odelström A., Adill A., Aho T., Jutfelt F. (2016). Physiological Constraints to Climate Warming in Fish Follow Principles of Plastic Floors and Concrete Ceilings. Nat. Commun..

[26] Larsson S. (2005). Thermal Preference of Arctic Charr, Salvelinus Alpinus, and Brown Trout, Salmo Trutta—Implications for Their Niche Segregation. Environ. Biol. Fish.

[27] Nivelle R., Gennotte V., Kalala E.J.K., Ngoc N.B., Muller M., Mélard C., Rougeot C. (2019). Temperature Preference of Nile Tilapia (*Oreochromis niloticus*) Juveniles Induces Spontaneous Sex Reversal. PLoS ONE.

[28] Rahmann H., Hilbig R. (1981). The Possible Functional Role of Neuronal Gangliosides in Temperature Adaptation of Vertebrates. J. Therm. Biol..

[29] Logue J.A., De Vries A.L., Fodor E., Cossins A.R. (2000). Lipid Compositional Correlates of Temperature-Adaptive Interspecific Differences in Membrane Physical Structure. J. Exp. Biol..

[30] Ernst R., Ejsing C.S., Antonny B. (2016). Homeoviscous Adaptation and the Regulation of Membrane Lipids. J. Mol. Biol..

[31] Malekar V.C., Morton J.D., Hider R.N., Cruickshank R.H., Hodge S., Metcalf V.J. (2018). Effect of Elevated Temperature on Membrane Lipid Saturation in Antarctic Notothenioid Fish. PeerJ.

[32] Maffioli E., Nonnis S., Negri A., Fontana M., Frabetti F., Rossi A.R., Tedeschi G., Toni M. (2024). Environmental Temperature Variation Affects Brain Lipid Composition in Adult Zebrafish (*Danio rerio*). Int. J. Mol. Sci..

[33] Rahmann H., Jonas U., Kappel T., Hildebrandt H. (1998). Differential Involvement of Gangliosides versus Phospholipids in the Process of Temperature Adaptation in Vertebrates: A Comparative Phenomenological and Physicochemical Study. Ann. N. Y. Acad. Sci..

[34] Rahmann H. (1990). Gangliosides as Modulators of Synaptic Transmission and Long-Term Neuronal Adaptation. Trends Glycosci. Glycotechnol..

[35] Avrova N.F. (2021). Brain Gangliosides and Their Functions as Natural Adaptogens. Neurosci. Behav. Phys..

[36] Kolter T., Proia R.L., Sandhoff K. (2002). Combinatorial Ganglioside Biosynthesis. J. Biol. Chem..

[37] Prinetti A., Loberto N., Chigorno V., Sonnino S. (2009). Glycosphingolipid Behaviour in Complex Membranes. Biochim. Biophys. Acta.

[38] Simons K., Toomre D. (2000). Lipid Rafts and Signal Transduction. Nat. Rev. Mol. Cell Biol..

[39] Pike L.J. (2006). Rafts Defined: A Report on the Keystone Symposium on Lipid Rafts and Cell Function. J. Lipid Res..

[40] Vernier P., Kaas J.H. (2017). 1.04—The Brains of Teleost Fishes. Evolution of Nervous Systems.

[41] Nieuwenhuys R., Donkelaar H.J., Nicholson C. (1998). The Central Nervous System of Vertebrates.

[42] Becker K., Wöhrmann A.P.A., Rahmann H. (1995). Brain Gangliosides and Cold-Adaptation in High-Antarctic Fish. Biochem. Syst. Ecol..

[43] Behan-Martin M.K., Jones G.R., Bowler K., Cossins A.R. (1993). A near Perfect Temperature Adaptation of Bilayer Order in Vertebrate Brain Membranes. Biochim. Biophys. Acta.

[44] Gagneux P., Panin V., Hennet T., Aebi M., Varki A., Varki A., Cummings R.D., Esko J.D., Stanley P., Hart G.W., Aebi M., Mohnen D., Kinoshita T., Packer N.H., Prestegard J.H. (2022). Evolution of Glycan Diversity. Essentials of Glycobiology.

[45] Rahmann H., Hilbig R. (1983). Phylogenetical Aspects of Brain Gangliosides in Vertebrates. J. Comp. Physiol. B.

[46] Hilbig R., Rahmann H., Rösner H. (1979). Brain Gangliosides and Temperature Adaptation in Eury- and Stenothermic Teleost Fish (Carp and Rainbow Trout). J. Therm. Biol..

[47] Kappel T., Anken R.H., Hanke W., Rahmann H. (2000). Gangliosides Affect Membrane-Channel Activities Dependent on Ambient Temperature. Cell. Mol. Neurobiol..

[48] Zehmer J.K., Hazel J.R. (2005). Thermally Induced Changes in Lipid Composition of Raft and Non-Raft Regions of Hepatocyte Plasma Membranes of Rainbow Trout. J. Exp. Biol..

[49] van Echten G., Sandhoff K. (1989). Modulation of Ganglioside Biosynthesis in Primary Cultured Neurons. J. Neurochem..

[50] Avrova N.F. (1985). Evolutionary Approach to the Analysis of Structure and Function of Gangliosides. Neurochem. Res..

[51] Hilbig R., Rahmann H. (1980). Variability in Brain Gangliosides of Fishes. J. Neurochem..

[52] Viljetić B., Labak I., Majić S., Stambuk A., Heffer M. (2012). Distribution of Mono-, Di- and Trisialo Gangliosides in the Brain of Actinopterygian Fishes. Biochim. Biophys. Acta.

[53] Bǎnǎrescu P.M., Paepke H.-J., The Freshwater Fishes of Europe (2001). Cyprinidae 2, Gasterosteidae.

[54] Stanković D., Crivelli A.J., Snoj A. (2015). Rainbow Trout in Europe: Introduction, Naturalization, and Impacts. Rev. Fish. Sci. Aquac..

[55] Schnaar R.L. (1994). Isolation of Glycosphingolipids. Guide to Techniques in Glycobiology.

[56] Svennerholm L. (1957). Quantitive Estimation of Sialic Acids: II. A Colorimetric Resorcinol-Hydrochloric Acid Method. Biochim. Biophys. Acta.

[57] Schneider C.A., Rasband W.S., Eliceiri K.W. (2012). NIH Image to ImageJ: 25 Years of Image Analysis. Nat. Methods.

[58] Schnaar R.L., Needham L.K. (1994). Thin-Layer Chromatography of Glycosphingolipids. Meth. Enzymol..

[59] BioRender.com. https://app.biorender.com/illustrations/65fb12682d08c19562fd5ba5.

[60] Armstrong J.B., Fullerton A.H., Jordan C.E., Ebersole J.L., Bellmore J.R., Arismendi I., Penaluna B., Reeves G.H. (2021). The Importance of Warm Habitat to the Growth Regime of Cold-Water Fishes. Nat. Clim. Chang..

[61] Moore M.J., Paukert C.P., Moore T.L. (2021). Effects of Latitude, Season, and Temperature on Lake Sturgeon Movement. N. Am. J. Fish. Manag..

[62] Popović J. (1994). Značajke Ljuske Ilirskog Klena. Ribarstvo.

[63] Raleigh R.F., Hickman T., Solomon R.C., Nelson P.C. (1984). Habitat Suitability Information: Rainbow Trout.

[64] Balon E.K. (1995). Origin and Domestication of the Wild Carp, *Cyprinus carpio*: From Roman Gourmets to the Swimming Flowers. Aquaculture.

[65] Topal A., Özdemir S., Arslan H., Çomaklı S. (2021). How Does Elevated Water Temperature Affect Fish Brain? (A Neurophysiological and Experimental Study: Assessment of Brain Derived Neurotrophic Factor, cFOS, Apoptotic Genes, Heat Shock Genes, ER-Stress Genes and Oxidative Stress Genes). Fish Shellfish Immunol..

[66] Li Q., Xiong L., Zhu Y., Zheng A., Zheng S. (2024). Effects of Acute Temperature Stress on the Expression of Related Genes in the Brain of *Opsariichthys bidens*. Fishes.

[67] Su J., Li W., Li H., Zhou Z., Miao Y., Yuan Y., Li Y., Tao M., Zhang C., Zhou Y. (2024). Comparative Transcriptomic Analysis of the Brain-Liver Axis Reveals Molecular Mechanisms Underlying Acute Cold Stress Response in Gynogenetic Mrigal Carp. Aquaculture.

[68] Yang Y., Yu H., Li H., Wang A., Yu H. (2016). Effect of High Temperature on Immune Response of Grass Carp (*Ctenopharyngodon idellus*) by Transcriptome Analysis. Fish Shellfish Immunol..

[69] Haesemeyer M. (2020). Thermoregulation in Fish. Mol. Cell. Endocrinol..

[70] Rahmann H. (1978). Gangliosides and Thermal Adaptation in Vertebrates. Jpn. J. Exp. Med..

[71] Probst W., Rösner H., Wiegandt H., Rahmann H. (1979). The Complexing Ability of Gangliosides for Ca2, I. Influence of Mono- and Divalent Cations and of Acetylcholin (Author’s transl). Hoppe-Seyler’s Z. Physiol. Chem..

[72] Probst W., Rahmann H. (1980). Influence of Temperature Changes on the Ability of Gangliosides to Complex with Ca^2+^. J. Therm. Biol..

[73] Rösner H., Breer H., Hilbig R., Rahmann H. (1979). Temperature Effects on the Incorporation of Sialic Acid into Gangliosides and Glycoproteins of Fish Brain. J. Therm. Biol..

[74] van den Burg E.H., Verhoye M., Peeters R.R., Meek J., Flik G., Van der Linden A. (2006). Activation of a Sensorimotor Pathway in Response to a Water Temperature Drop in a Teleost Fish. J. Exp. Biol..

[75] van den Burg E.H., Peeters R.R., Verhoye M., Meek J., Flik G., Van der Linden A. (2005). Brain Responses to Ambient Temperature Fluctuations in Fish: Reduction of Blood Volume and Initiation of a Whole-Body Stress Response. J. Neurophysiol..

[76] Kristal B.S., Brown A.M. (1999). Apoptogenic Ganglioside GD3 Directly Induces the Mitochondrial Permeability Transition. J. Biol. Chem..

[77] Dhanushkodi A., Xue Y., Roguski E.E., Ding Y., Matta S.G., Heck D., Fan G.-H., McDonald M.P. (2019). Lentiviral-Mediated Knock-down of GD3 Synthase Protects against MPTP-Induced Motor Deficits and Neurodegeneration. Neurosci. Lett..

[78] Inamori K., Inokuchi J. (2020). Roles of Gangliosides in Hypothalamic Control of Energy Balance: New Insights. Int. J. Mol. Sci..

[79] Tseng Y.-C., Liu S.-T., Hu M.Y., Chen R.-D., Lee J.-R., Hwang P.-P. (2014). Brain Functioning under Acute Hypothermic Stress Supported by Dynamic Monocarboxylate Utilization and Transport in Ectothermic Fish. Front. Zool..

[80] Bueschke N., do Amaral-Silva L., Hu M., Santin J.M. (2021). Lactate Ions Induce Synaptic Plasticity to Enhance Output from the Central Respiratory Network. J. Physiol..

[81] Soengas J.L., Aldegunde M. (2002). Energy Metabolism of Fish Brain. Comp. Biochem. Physiol. Part B Biochem. Mol. Biol..

[82] Cai M., Wang H., Song H., Yang R., Wang L., Xue X., Sun W., Hu J. (2022). Lactate Is Answerable for Brain Function and Treating Brain Diseases: Energy Substrates and Signal Molecule. Front. Nutr..

[83] Wullimann M.F., Meyer D.L. (1990). Phylogeny of Putative Cholinergic Visual Pathways through the Pretectum to the Hypothalamus in Teleost Fish. Brain Behav. Evol..

[84] Kanwal J.S., Finger T.E., Caprio J. (1988). Forebrain Connections of the Gustatory System in Ictalurid Catfishes. J. Comp. Neurol..

[85] de Bruin J.P.C., Ebbesson S.O.E. (1980). Telencephalon and Behavior in Teleost Fish. Comparative Neurology of the Telencephalon.

[86] Wullimann M.F., Northcutt R.G. (1988). Connections of the Corpus Cerebelli in the Green Sunfish and the Common Goldfish: A Comparison of Perciform and Cypriniform Teleosts. Brain Behav. Evol..

[87] Hilbig R. (1983). Regional Differences in Brain Gangliosides of a Teleost Fish Following Thermal Acclimations. J. Therm. Biol..

[88] Rösner H. (2003). Developmental Expression and Possible Roles of Gangliosides in Brain Development. Prog. Mol. Subcell. Biol..

[89] Yu R.K., Macala L.J., Taki T., Weinfeld H.M., Yu F.S. (1988). Developmental Changes in Ganglioside Composition and Synthesis in Embryonic Rat Brain. J. Neurochem..

[90] Qi Y., Xue Q. (1991). Ganglioside Levels in Hypoxic Brains from Neonatal and Premature Infants. Mol. Chem. Neuropathol..

[91] Britton J.R., Boar R.R., Grey J., Foster J., Lugonzo J., Harper D.M. (2007). From Introduction to Fishery Dominance: The Initial Impacts of the Invasive Carp *Cyprinus carpio* in Lake Naivasha, Kenya, 1999 to 2006. J. Fish Biol..

[92] Miller S.A., Provenza F.D. (2007). Mechanisms of Resistance of Freshwater Macrophytes to Herbivory by Invasive Juvenile Common Carp. Freshw. Biol..

[93] Pinto L., Chandrasena N., Pera J., Hawkins P., Eccles D., Sim R. (2005). Managing Invasive Carp (*Cyprinus carpio* L.) for Habitat Enhancement at Botany Wetlands, Australia. Aquat. Conserv. Mar. Freshw. Ecosyst..

[94] Yeagle P.L. (1989). Lipid Regulation of Cell Membrane Structure and Function. FASEB J..

[95] Sandhoff R., Sandhoff K. (2018). Emerging Concepts of Ganglioside Metabolism. FEBS Lett..

[96] Yu R.K., Ariga T., Yanagisawa M., Zeng G., Fraser-Reid B.O., Tatsuta K., Thiem J. (2008). Gangliosides in the Nervous System: Biosynthesis and Degradation. Glycoscience: Chemistry and Chemical Biology.

[97] Spessott W., Crespo P.M., Daniotti J.L., Maccioni H.J.F. (2012). Glycosyltransferase Complexes Improve Glycolipid Synthesis. FEBS Lett..

[98] Lauc G., Dabelic S., Supraha S., Flögel M. (1998). Glycobiology of Stress. Ann. N. Y. Acad. Sci..

[99] Nakayama J., Fukuda M.N., Hirabayashi Y., Kanamori A., Sasaki K., Nishi T., Fukuda M. (1996). Expression Cloning of a Human GT3 Synthase. J. Biol. Chem..

[100] Schnaar R.L., Gerardy-Schahn R., Hildebrandt H. (2014). Sialic Acids in the Brain: Gangliosides and Polysialic Acid in Nervous System Development, Stability, Disease, and Regeneration. Physiol. Rev..

[101] McDonald A.G., Hayes J.M., Davey G.P. (2016). Metabolic Flux Control in Glycosylation. Curr. Opin. Struct. Biol..

[102] Desplats P.A., Denny C.A., Kass K.E., Gilmartin T., Head S.R., Sutcliffe J.G., Seyfried T.N., Thomas E.A. (2007). Glycolipid and Ganglioside Metabolism Imbalances in Huntington’s Disease. Neurobiol. Dis..

[103] Roy J., Vigor C., Vercauteren J., Reversat G., Zhou B., Surget A., Larroquet L., Lanuque A., Sandres F., Terrier F. (2020). Characterization and Modulation of Brain Lipids Content of Rainbow Trout Fed with 100% Plant Based Diet Rich in Omega-3 Long Chain Polyunsaturated Fatty Acids DHA and EPA. Biochimie.

