# Supplementary material for: Temperature-Induced Seasonal Dynamics of Brain Gangliosides in Rainbow Trout (Oncorhynchus mykiss Walbaum) and Common Carp (Cyprinus carpio L.)
Source: Life (Basel). 2024 Oct 7;14(10):1273. doi: 10.3390/life14101273 (PMC11509357; doi:10.3390/life14101273)
Supplement: Supplementary file 1 [file life-14-01273-s001.zip › life-3176701-supplementary/S_Tables.pdf]

Table S1. Measured values of water temperature (Tw) and air (Ta) and dissolved oxygen (O_2) concentration in water at 9h during the experimental period for rainbow trout, location Turnić, Požega, indicated sampling days.

January	Ta in 9h / °C	Tw/ °C	O_2/ mg L^{-1}	February	Ta in 9h / °C	Tw/ °C	O_2/ mg L^{-1}	March	Ta in 9h / °C	Tw/ °C	O_2/ mg L^{-1}
1	-2	10,8	10,8	1	4	11,5	9,1	1	3,7	10	9,9
2	-4	10,9	10	2	4	11,4	9,2	2	3	11,2	9
3	-2	10,7	10,8	3	0	11	9,3	3	3,5	11,1	9
4	-3	10,5	10,6	4	0	11	9,3	4	3	11,3	9,1
5	1	10,9	10,4	5	0,5	11	9,2	5	4	11	9,5
6	0	10,7	10,4	6	1	11,2	9,1	6	1	11,1	9,4
7	0	11	10,4	7	2	11	9	7	1	11	9,2
8	-1	11,1	10,2	8	2,5	10	9,5	8	5	11	9,6
9	3	11	10,3	9	3	11,2	9	9	4	11	9,8
10	1	11	10,3	10	3	11,1	9,5	10	3	11,2	9,9
11	0	11	10,2	11	3,5	11,3	9	11	0	10	10,5
12	1	11,2	8	12	2	11	9	12	4	11,2	9
13	0	11	9,8	13	2	11,1	9,1	13	5	11,1	9
14	-2	10	9,9	14	3	11	9,5	14	6	11,3	9,1
15	1	11,2	10	15	3	11	9,4	15	6	11	9,5
16	3	11,1	9,8	16	2,5	11	9,2	16	7	11,1	9,4
17	3	11,3	9,4	17	3	11,2	9,6	17	5	10	10,4
18	5,6	11,6	9,1	18	3	11	9,8	18	4	11,2	9,8
19	6	11	9,6	19	4	11,1	9,9	19	2	10	10
20	6	11,5	9,4	20	3,9	11	10	20	5	11,3	10
21	4	11,5	9,1	21	2	11	10	21	4	11	10

22	4	11,4	9	22	2	11	9,9	22	6	11,1	10
23	3	11,6	9	23	1	11,2	9,6	23	7	11	10,2
24	7	11,5	9,6	24	2	11	9,9	24	7,5	11	10,3
25	4	11,6	8,9	25	4	11,2	10	25	6,3	11	10,4
26	5	11,7	8,8	26	4	11,3	10,6	26	6,5	11,2	9,8
27	5	11,8	8,9	27	3	11,1	10,8	27	5,9	10	10
28	4	11,6	8,7	28	2	11	10,9	28	7	11,3	9,6
29	5	11,4	8,9	29	3	11	11	29	7,3	11	9,4
30	5	11,6	8,7		-	-	-	30	8	11,1	8,9
31	7	11,9	8,9		-	-	-	31	8,5	11,4	8,9

**April**	**Ta in 9h / °C**	**Tw/ °C**	**O_2/ mg L^{-1}**	**May**	**Ta in 9h / °C**	**Tw/ °C**	**O_2/ mg L^{-1}**	**June**	**Ta in 9h / °C**	**Tw/ °C**	**O_2/ mg L^{-1}**
1	7	11,2	9	1	11	11,6	9,1	1	15	10,5	8,4
2	7,8	11,1	9	2	11,5	11,5	9,2	2	16	11,6	8,2
3	7,9	11,3	9,1	3	11,9	11,9	9,4	3	16,5	11,8	8,1
4	7,9	11	9,5	4	12	11,6	10,4	4	16,4	11,5	8,4
5	8	11,1	9,4	5	12	11,5	9,8	5	17	11,6	8,4
6	8	10	10,4	6	11	11,6	9,1	6	17,5	11,6	8,3 *
7	9	11,2	9,8	7	11,5	11,5	9,2	7	15	10,5	7,5
8	3	10	10	8	11,9	11,9	9,4	8	16	11,6	7,2
9	8	11,3	10	9	12	11,6	10,4	9	18	11,8	7,1
10	9	11	10	10	13	11,5	10,2	10	16	11,8	7,1
11	9	11,1	10	11	10	11	10	11	17	11,6	7,4
12	10	11	10,2	12	5	10	10,3	12	17,5	11,6	7,3
13	9	11,2	9,5	13	10	11	10,2	13	15	10,5	7,5

14	7	11	9,6	14	9	11,2	9,1	14	16	11,6	7,2
15	11	11,6	9,1	15	8	11	9	15	18	11,8	7,1
16	11,5	11,5	9,2	16	13	11,5	8,9	16	16	12,1	7,1
17	11,9	11,9	9,4	17	10	11	9,5	17	17,9	12,2	6,9
18	12	11,6	10,4	18	14,5	11,5	8,4	18	17,5	11,6	7,3 **
19	12	11,5	9,8	19	17	12	8,5	19	15	10,5	8,5
20	11	11,2	10	20	11	10	9,1	20	18	11,8	8,1
21	10	11,1	10	21	13	10,3	9	21	16	11,8	8,1
22	12,6	11,6	10	22	11	10,2	9,4	22	17	11,6	8,4
23	13	11,5	10,2	23	11,2	9,1	9,5	23	17,5	11,6	8,8
24	10	11	10	24	13	10	8,4	24	15	10,5	8,8
25	5	10	10,3	25	14	10,5	8,5	25	16	11,6	8,6
26	10	11	10,2	26	15	10,5	8,4	26	18	12,1	8,1
27	9	11,2	9,5	27	16	11,6	8,2	27	12	11	9
28	7	11	9,6	28	16,5	11,8	8,1	28	10	11	10
29	10	11	10,2	29	16,4	11,5	8,4	29	16	11,5	9,4
30	9	11,2	9,8	30	17	11,6	8,4	30	18	11,8	9,1
	-	-	-	31	17,5	11,6	8,3		-	-	-

*- introduced young fish into the water system

**- turning on the aerator

Table S2. Measured values of water temperature (Tw) and air (Ta) and dissolved oxygen (O_2) concentration in water at 9h during the experimental period for common carp, **location Grudnjak, Orahovica,** indicated sampling days.

January	Ta in 9h / °C	Tw/ °C	O_2/ mg L^{-1}	February	Ta in 9h / °C	Tw/ °C	O_2/ mg L^{-1}	March	Ta in 9h / °C	Tw/ °C	O_2/ mg L^{-1}
1	-3	6	13	1	10	5	12,5	1	2	6,5	12
2	-3,5	5,8	13	2	10	4,9	12,2	2	2	6,5	12
3	-2	5	12,8	3	6	5	12	3	1	6,3	12,1
4	0	5,1	12,5	4	5	6	11,7	4	2	6,5	12
5	3	5	12,4	5	2	6	12	5	3	6,5	12,2
6	3	5	12,4	6	3	5	12	6	2	6,5	12
7	3	5	12,4	7	4	4,5	12,6	7	-1	6	12,2
8	3	5	12,4	8	6	5	12	8	2	6,5	12
9	3	6,1	12	9	4	5	11,7	9	2	6,5	12
10	3	5	12,1	10	2	6	12	10	2	6,5	12
11	3	6	12	11	3	5	12	11	3	6,5	12
12	-3	4,4	12,6	12	4	4,5	12,6	12	2	6,5	12
13	-1	3,7	13,7	13	3	5	12	13	3	7	11,7
14	-1	3,7	13,6	14	3	5	12	14	4	8	11,5
15	-1	3,6	13,7	15	4	5,2	12	15	4	8,2	11,5
16	2	4,5	13,2	16	3	5	12,1	16	4,5	8	11,3
17	2	4,5	13,2	17	3	5	12,3	17	5	8,5	11,5
18	2	4,5	13,2	18	2	5,1	12	18	4	8	11,3
19	2	4,5	11,6	19	0	3,5	12,5	19	4	8	11,2
20	2	4,3	12	20	2	5	12	20	4	8	11,5
21	2	4,5	12,1	21	0	5	11,8	21	7	10	11

22	0	4,1	12	22	-1	5	11,9	22	7	10	11,2
23	2	4,5	11,5	23	0	3,5	12,5	23	7	10,5	11,1
24	2	4,5	11,5	24	2	5	12	24	7	10,5	11
25	0	3	13,5	25	0	3,5	12,5	25	7	10	11
26	2	4,5	12,3	26	2	5	12	26	7	10	11,2
27	0	3	13,5	27	0	5	11,8	27	7,5	10,5	11,1
28	4	3,3	12,7	28	1	6	12	28	8	11,5	11
29	4	3,3	12,5	29	2	6,5	12,4	29	7	10	11
30	6	4	12,5		-	-	-	30	7	10	11,2
31	10	5,5	11,5		-	-	-	31	7	10,5	11,1
**April**	**Ta in 9h / °C**	**Tw/ °C**	**O_2/ mg L^{-1}**	**May**	**Ta in 9h / °C**	**Tw/ °C**	**O_2/ mg L^{-1}**	**June**	**Ta in 9h / °C**	**Tw/ °C**	**O_2/ mg L^{-1}**
1	7,5	10,5	11	1	8	14	11	1	20	21,5	9,8
2	7	10,5	11,2	2	8	16	10,7	2	21	22	9,6
3	8	11,5	11,1	3	7	17	10,5	3	21	20,5	9,7
4	8	12,5	10,9	4	8	16	10,9	4	23	21,5	9,6
5	8	11,5	11,1	5	1	11	12	5	19	20,5	10
6	8	12,5	10,9	6	5	11	11,5	6	19	20,5	10
7	8	12,5	11	7	10	12	11,5	7	21	22	9,8
8	8	12,5	10,9	8	15	14	10,8	8	21	22	9,6
9	9	12,5	11	9	13	16	10,6	9	21	21,5	9,4
10	10	13	11	10	13	16,5	11	10	18	21,5	9,4
11	10	13,5	10,8	11	13	18	11	11	17	20,5	9,5
12	9	12,5	11	12	15	18,5	10,8	12	16	20,5	9,4
13	10	13	11	13	13	16,5	11	13	19	20	9,8

14	10	13,5	10,8	14	13	18	11	14	23	22,5	9,4
15	9	12,5	11	15	15	18,5	10,8	15	24	22	9,2
16	10	13	11	16	13	16,5	11	16	25	22,5	9,2
17	5	12	11,6	17	13	16,5	11	17	27	24,5	9,5
18	4	12	11,8	18	15	18	11	18	22	23,5	9,3
19	8	14	11,3	19	15	18,5	10,8	19	23	23,5	9,2
20	8	11,5	11,1	20	17	19	10,5	20	16	20,5	9,8
21	8	12,5	10,9	21	18	20	10,4	21	19	21,5	9,6
22	8	12,5	11	22	20	20,5	10	22	23	22,5	9,3
23	8	12,5	10,9	23	21	20,5	10,2	23	26	23,5	9,1
24	9	12,5	11	24	21	21	9,8	24	22	23,5	9,5
25	8	11,5	11,1	25	23	21	10	25	20	21,5	9,4
26	10	13	10,8	26	25	22	9,6	26	22	22,5	9,6
27	10	13,2	10,8	27	21	21,5	10,2	27	20	23	9,8
28	10	13	10,8	28	23	21,5	10	28	16	20	10,2
29	10	13,2	10,8	29	24	22	9,6	29	19	21	9,6
30	11	13,5	10,7	30	21	22,5	10,2	30	23	22	9,5
	-	-	-	31	23	22,5	10		-	-	-
