# Supplementary figures and images for: Temperature-Induced Seasonal Dynamics of Brain Gangliosides in Rainbow Trout (Oncorhynchus mykiss Walbaum) and Common Carp (Cyprinus carpio L.)
Source: Life (Basel). 2024 Oct 7;14(10):1273. doi: 10.3390/life14101273 (PMC11509357; doi:10.3390/life14101273)

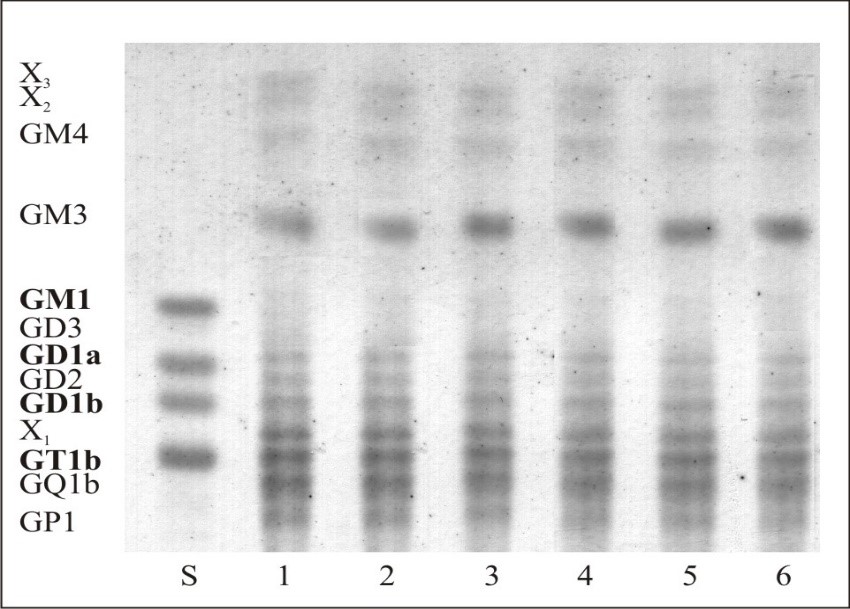

Supplement: Supplementary file 1 [file life-14-01273-s001.zip › life-3176701-supplementary/Figure S1.jpg]

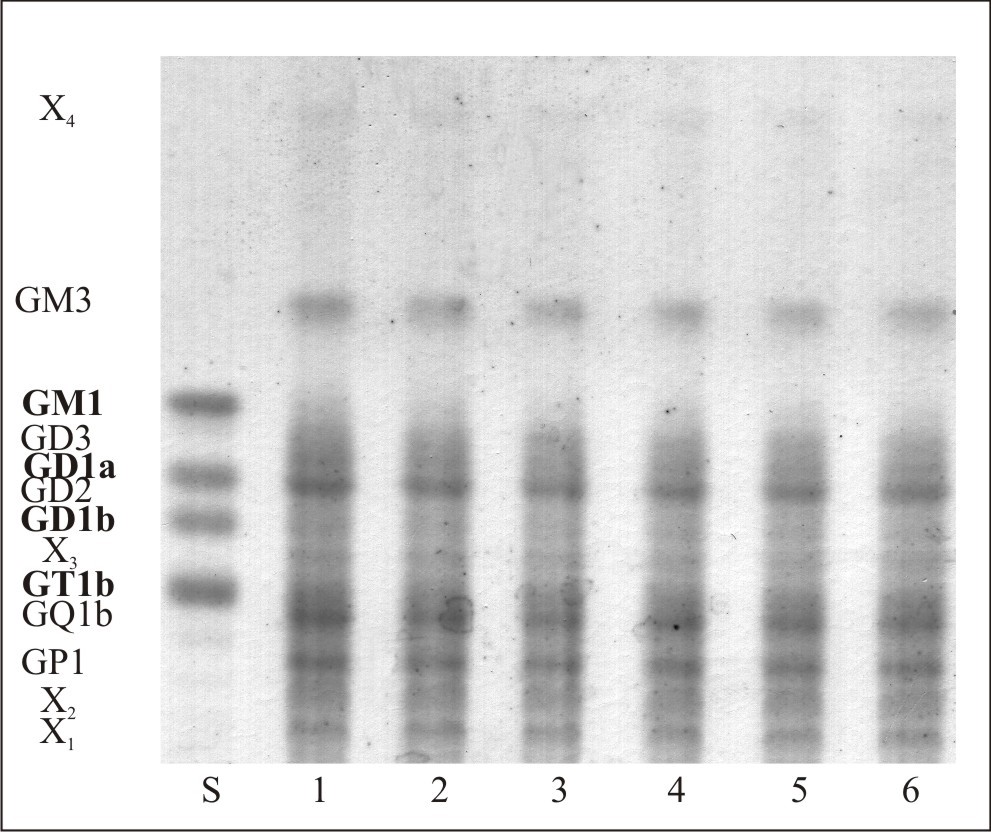

Supplement: Supplementary file 1 [file life-14-01273-s001.zip › life-3176701-supplementary/Figure S2.jpg]
